# Cognitive training using the abacus: a literature review study on the benefits for different age groups

**DOI:** 10.1590/1980-57642021dn15-020014

**Published:** 2021

**Authors:** Thais Bento Lima-Silva, Maurício Einstoss de Castro Barbosa, Mariana Garcia Zumkeller, Cássia Elisa Rosseto Verga, Patrícia Lessa Prata, Neide Pereira Cardoso, Luiz Carlos de Moraes, Sonia Maria Dozzi Brucki

**Affiliations:** 1 Group of Cognitive and Behavioral Neurology, Hospital das Clínicas, Faculdade de Medicina, Universidade de São Paulo - São Paulo, SP, Brazil.; 2 School of Arts, Science and Humanities, Universidade de São Paulo - São Paulo, SP, Brazil.; 3 Instituto Supera de Educação - São José dos Campos, SP, Brazil.

**Keywords:** mental health, cognition, aging, working memory, spatial memory, executive function, saúde mental, cognição, envelhecimento, memória operacional, memória espacial, função executiva

## Abstract

The literature indicates that cognitive stimulation interventions have shown promising results. Abacus represents a tool with great potential in such interventions.

**Objectives:**

To carry out a systematic review of studies published in recent years that entailed the delivery of a cognitive training program using an abacus to boost target cognitive abilities of older persons and also other age groups, with or without cognitive impairment.

**Methods:**

A systematic review study was conducted in July 2020 involving PubMed, MedLine, LILACS, and SciELO databases.

**Results:**

A total of 29 studies were retrieved, of which 8 aimed to identify the effect of abacus-based mental calculation (AMC) for different age groups and to determine its applicability as a method of cognitive stimulation for older adults. In AMC technique, participants first learn to use the physical abacus (PA) and after achieving proficiency they perform calculations using a mental image of the device, manipulating the beads of the so-called mental abacus (MA).

**Conclusions:**

The number of studies addressing abacus use as a cognitive training tool was rather limited, considering the relevance of the theme. Their interventions have shown benefits for cognitive functioning of individuals of various age groups, including older adults with cognitive impairment. Future studies that involve larger samples of healthy and/or cognitively impaired older adults with a longitudinal design and a more elaborate methodological design are suggested.

## RESUMO.

A literatura aponta que intervenções de estimulação cognitiva têm mostrado resultados promissores. O ábaco representa uma ferramenta com grande potencial nesse tipo de intervenções.

**Objetivo:**

Realizar uma revisão sistemática de estudos publicados nos últimos anos que buscaram, em seus métodos, oferecer um programa de treino cognitivo com o uso do ábaco para estimular habilidades-alvo em idosos e em pessoas de outras faixas etárias, com ou sem comprometimento cognitivo.

**Métodos:**

Estudo de revisão sistemática, realizado em julho de 2020, utilizando-se as bases de dados PubMed, MedLine, LILACS e SciELO.

**Resultados:**

Um total de 29 estudos foram encontrados, dos quais oito objetivaram identificar o efeito do treino de uma técnica chamada *abacus-based mental calculation* (AMC) em diferentes faixas etárias e verificar sua aplicabilidade como método de estimulação cognitiva para idosos. No AMC, os participantes aprendem inicialmente a manipular o ábaco físico (PA, na sigla em inglês) e, após adquirirem agilidade, passam a realizar cálculos com uma imagem mental do instrumento, manipulando as contas do denominado ábaco mental (MA, na sigla em inglês).

**Conclusões:**

O número de estudos que abordaram o uso do ábaco como ferramenta para realização de treino cognitivo foi bastante limitado diante da relevância do tema. Os estudos analisados apresentaram benefícios do treino cognitivo com uso do ábaco para o desempenho cognitivo de indivíduos de diversas faixas etárias, inclusive em idosos com comprometimento cognitivo. Para estudos futuros, espera-se a realização de mais pesquisas com amostras maiores, com delineamento longitudinal e com métodos bem elaborados, com enfoque em pessoas idosas, saudáveis e/ou com comprometimento cognitivo.

## INTRODUCTION

According to the theory behind the concept of cognitive reserve, greater brain activity can slow the onset of cognitive impairment, even in extremely old people.^1^ ^,^ ^2^ Aguirre et al.^3^ and McDermott et al.,^4^ systematic review and meta-analysis studies, have shown that cognitive stimulation interventions have favourable results on cognitive and physical functions, social interaction, activities of daily living, quality of life and well-being of individuals with dementia, with the possibility of overcoming effects of medicinal products.

Cognitive stimulation is a type of non-pharmacological educational intervention aimed at enhancing cognitive functioning in cognitively healthy individuals and subjects with mild cognitive impairment (MCI) through the use of mnemonic strategies. These interventions include theory and practice with cognitive exercises to promote improved performance, and can have positive effects on the everyday lives of older adults.^5^ Trained individuals can show greater health promotion, improved quality of life and less deficits in Instrumental Activities of Daily Living (IADLs).^6^ ^,^ ^7^

Working memory (WM) is a cognitive mechanism defined as a short-term memory subsystem with the capacity to store and process data simultaneously in a short period of time.^8^ Thus, WM represents a dynamic capacity which works like a system for retention, retrieval and temporary manipulation of multiple items of information that serves as a basis for executing complex tasks. In order to function, WM recruits areas such as the pre-frontal cortex, hippocampus and also the entorhinal cortex, superior parietal lobules and anterior cingulate cortex.^9^ These areas are affected by aging, and this process can lead to impaired performance of WM tasks in older adults.^10^ ^,^ ^11^ ^,^ ^12^ ^,^ ^13^

Karbach and Verhaeghen^14^ carried out a meta-analysis of 49 articles assessing the effects of WM training in young and older adults. The authors showed that an intervention centering on WM had a positive impact in the group that underwent training relative to the control group, for both target tasks and proximal transfer tasks. On these tasks, the experimental group displayed better performance than both the control and active control groups. Regarding distal transfer tests, benefits were also seen for the experimental group *versus* the control group in fluid intelligence, episodic memory, attention, inhibitory control and processing speed, but training effects were less marked for processing speed.

Another meta-analysis, by Schwaighofer et al.,^15^ encompassing 47 articles, investigated the effect of different factors on the outcome of WM training. To this end, WM interventions were performed in a cohort aged from 4 to 71 years. The authors demonstrated that the factors age, number of training days per week, time between sessions, modality (group or individual), and absence or presence of feedback were not associated with the effectiveness of the training. Number of sessions, however, appeared to impact visuospatial short-term memory, and greater number of sessions was associated with better WM performance by participants. Another factor found to influence short-term memory, in this case verbal, was session duration, i.e. the longer the session, the better the performance on verbal short-term memory tests.

**Figure 1.**
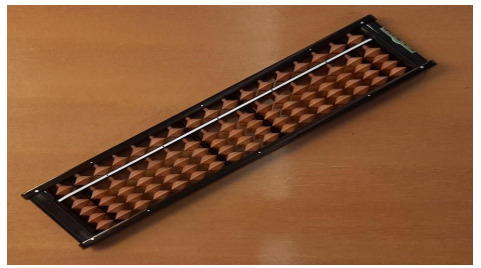
Image of the physical abacus, provided by the Instituto Supera de Educação, 2020.

**Figure 2.**
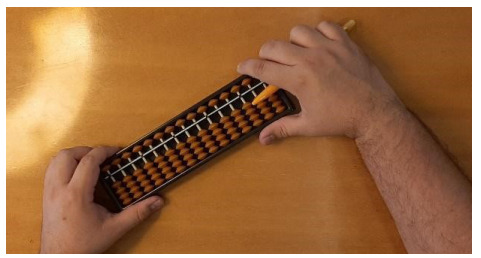
Image of a person using the physical abacus, provided by the Instituto Supera de Educação, 2020.

Some interventions have been run to document the benefits of abacus training in young children. These interventions aimed to stimulate attention and reasoning, as well as elucidate potential transfer effects to other cognitive abilities.^16^ ^,^ ^17^ The abacus is a kind of mechanical calculating device used in Eastern and Asian countries since 1200 AD to perform arithmetic, including addition, subtraction, multiplication, division and root calculations (see Figures 1 and 2). It represents numbers via an arrangement of beads in columns, each column representing a place value that increases from right to left. In long term cognitive training sessions, carried out in schools as Instituto Supera de Educação, which use the abacus as the main stimulus of cognitive training, progressively more complex calculations are carried out until the participant attains their maximum level and performance. The task is executed after undergoing stimuli of different cognitive abilities.^18^

Studies show that abacus training in children improves development of structural and functional changes, white matter volume, the parameter of white matter diffusion and activation patterns in cognitive tasks.^19^ In addition, studies on abacus training in children have shown evidence of benefits in learning performance, in the orientation ability of visuospatial attention WM, in attention and comprehensive arithmetic abilities, an also in response inhibition through neuroanatomical changes in areas governing these functions.^20^ ^,^ ^21^

In the literature, the pioneering study of Matías-Guiu et al.^22^ assessed a cognitive stimulation method based on abacus arithmetic calculations in the elderly population. Study participants aged ≥65 years were divided into 3 groups: healthy elderly (n=6), elderly with MCI (n=6) and elderly diagnosed with Alzheimer´s Disease (AD) (n=8). The results showed that participants had higher scores on the Mini-Mental State Examination (MMSE) after the intervention in 10 sessions of 150 minutes 2x per week with mixed groups (1 participant from each diagnostic group) of 10 participants comprising. However, no significant changes were evident in scores on tests measuring executive functions with the Trail-making Test A; selective attention with the Trail-making Test B; or depression symptoms with the Geriatric Depression Scale (GDS). The study results suggested that cognitive stimulation using the abacus can be useful in older adults with or without cognitive impairment.

In a literature review study^23^ investigating whether abacus training in humans promoted neural plasticity, the authors found that long-term abacus-based mental calculation (AMC) training affected calculation skills, WM and conceptual knowledge of the numeration system. Abacus training-induced functional and structural changes were located mainly in the right frontoparietal network and left fusiform gyrus areas.

It is known that the effects of training can extend to other domains, such as health promotion^24^ ^,^ ^25^ and functionality.^5^ ^,^ ^26^ When considering the findings presented and the impacts of the cognitive training performed with the use of the abacus for several domains in children, adults and older persons, it appears that participation in programs of this nature can favour psychological well-being and quality of life in old age, with an impact on safety and self-care. On the other hand, although the impacts of using an abacus as a cognitive training tool for the older persons are reported, research in this area is scarce. Thus, it is of utmost importance to demonstrate the findings verified so far as a way of encouraging future studies in this field, seeking to offer the population a promising strategy for promoting physical and mental health.

In this context, the objective of the present investigation was to carry out a systematic review of studies published in recent years that entailed the delivery of a cognitive training program using an abacus to boost target cognitive abilities of older persons and also other age groups, with or without cognitive impairment.

## METHOD

This is a systematic review research, carried out in July 2020. To guide the stages of identification, screening and eligibility of studies, we used the Statement of Preferred Reporting Items for Systematic Reviews and Meta-Analyzes (PRISMA)^27^ as a model. The initial identification of the studies was performed by searching PubMed, MedLine, LILACS and SciELO databases. In the screening, duplicate studies were excluded and titles and abstracts were read for the first selection, according to the pre-established inclusion and exclusion criteria. In the eligibility stage, the remaining studies were read in full in order to be selected according to the same criteria. The remaining studies after this stage were the studies included in the review.

Publications in English, Portuguese and Spanish were searched using the following descriptors and their respective equivalents: abacus, cognitive training, cognition and working memory. To this end, the following combinations were used: ("ábaco" OR "abacus") AND ("treino cognitivo" OR "cognitive training" OR "entrenamiento cognitivo"); ("ábaco" OR "abacus") AND ("cognição" OR "cognition" OR "cognición"); ("ábaco" OR "abacus") AND ("memória operacional" OR "working memory" OR "memoria operacional"). Due to the scarcity of studies identified in the initial search tests, it was decided not to determine a specific period of time, considering the studies regardless of the year of publication.

The searches and selection of studies included in this review were carried out by three researchers. After the completion of each stage (identification, screening and eligibility), the researchers met to jointly verify which studies would be included in this review. With regard to filters, we chose to select “full text”, to avoid records that contained only abstracts, and “randomized controlled trial”, according to the inclusion criteria adopted for this review.

The inclusion criteria were: to be a cognitive training study, to use the abacus as a tool for the interventions performed, to have presented training methods by age group in their methods (which could be cognitive training studies involving children, young people, adults, older adults and the elderly), and to present cognitive tools to measure the effectiveness of the training program offered. Exclusion criteria were: book chapters, doctoral theses, Masters dissertations, letters to editor, reflective essays and duplicate studies identified during the review. Articles with weak methods in terms of cognitive training protocol and measuring instruments used to determine the impact of the program on participants’ cognitive performance and the target abilities were also excluded.

During the analysis of the studies included in the review, we sought to assess their objectives, methods and results. The following data were observed: the use of the abacus as a training tool; the age range of the population studied; the impacts of using the abacus on cognitive functions, such as impacts on WM; the application of pre and post-tests; considerations regarding MCI; and the number of sessions held during the proposed intervention.

## RESULTS

**Figure 3.**
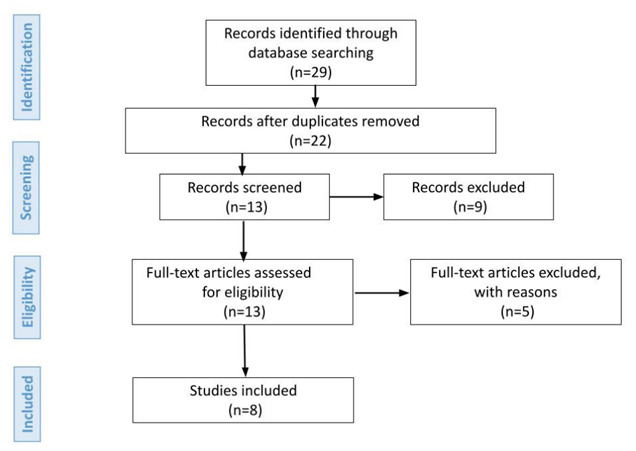
Flow diagram of process of study selection for inclusion in review.

The initial search retrieved 29 articles, further reduced to 22 after exclusion of duplicate studies. The titles and abstracts were read, and articles that failed to address the review objective were excluded. A total of 13 articles were read in full, of which 5 were subsequently excluded based on the study inclusion and exclusion criteria. Details of the study selection and inclusion process are shown in the flow diagram^27^ depicted in Figure 3.

Regarding samples, 6 studies involved children,^16^ ^,^ ^17^ ^,^ ^28^ ^,^ ^29^ ^,^ ^30^ ^,^ ^31^ 1 included young adults^32^ and 1 comprised older adults,^22^ with participant age ranging from 5 to 79 years. The objectives, methods and results of the 8 studies selected for analysis are charted in Table 1.

**Table 1.** Studies using abacus as a tool in cognitive training sessions.

Authors/Year	Objectives	Methods	Results found
Bhaskaran et al., 2006^31^	To evaluate short-term memory in children aged 5-12 years trained on the abacus for 1 and 2 years.	n=100 participants aged 5-12 years; intervention length: 2 years, 1h 2x week; study groups: (1) control group (n=50): no previous contact with abacus training; (2) AMC group (n=50): previously trained on abacus for 1 and 2 years; (3) control subgroup (n=20): followed for 2 years; (4) AMC subgroup (n=20): followed for 2 years; evaluated at baseline and after 1 and 2 years: Wechsler memory scale, MMSE, Brown Peterson Test, Mann - Buitar Visual Memory Screen for Objects.	Both control and AMC groups had better scores on tests, but AMC group showed more significant results. Improvements were observed for the AMC in short-term memory and visual retention memory, and also in visual and auditory memory.
Wang et al., 2015^29^	To explore the impact of long-term AMC training on math ability, task switching and the relationship between them.	n=82 participants (n=70 evaluated); children with mean age 6-7 years; intervention length: 3 years; study groups: (1) AMC group (n=31): 2h training per week; (2) control group (n=39): assessments at baseline and after 9 months and post-intervention: parent questionnaire, Raven Test, Go/No-go task, HRT, Dots task.	Results showed long-term AMC training was associated with better arithmetic and visuospatial abilities. An interaction between training and switch cost in predicting math abilities was also found, suggesting stronger associations between task switching and math abilities in AMC children.
Barner et al., 2016^28^	To test whether MA expertise (a) can be acquired in standard classroom settings, (b) improves students' mathematical abilities (beyond standard math curricula), and (c) is related to changes in basic cognitive capacities like working memory.	n=204 participants (n=183 analyzed); children with mean age 5-7 years; intervention length: 3 years; study groups: (1) MA group (n=100): 2 sessions of 90 min. per week of MA instruction (1 year on PA and 2 years on MA); (2) control group (n=104): 2 sessions of 90 min per week of supplemental mathematics training from standard curriculum; 1 test at baseline + 3 tests, one for each complete year of study: large battery of computerized and paper-based tasks including mathematics and general cognitive measures.	MA students outperformed controls on arithmetic tasks, suggesting that MA expertise can be achieved by children in standard classrooms. MA training did not alter basic cognitive abilities; instead, differences in SWM at the beginning of the study mediated MA learning.
Wang et al., 2017^17^	To investigate the effects of AMC training on the development of EF and its underlying brain activity.	n=72 participants (n=51 analyzed); children from first year of primary school; intervention length: 6 years; study groups: (1) AMC group: 2h per week of training; (2) control group: received extra learning on such materials as reading, traditional calculation, simple geometry, or some activities such as sports; evaluations at baseline and after 26 and 40 months; Combined Raven test, Go/no-go task, Dimensions of Mastery questionnaires, Early School Behavior Rating Scale, EF and fMRI task only in second and third testing sessions.	AMC-trained children were faster and more accurate than their peers, particularly in incongruent and contradictory conditions. Results suggest that the effects of AMC training on EF may not be solely limited to enhanced working memory, but also tap the inhibition and task switching components of EF. From the age of 10 to12 years, AMC-trained children showed activation decreases in frontoparietal regions while control children exhibited the opposite pattern.
Xie et al., 2018^30^	To determine whether and after how long AMC training reorganizes brain networks using graph theory.	n=162 participants; children with mean age 9-10 years; intervention length: 1 year: study groups: (1) AMC group (n=90) 2h per week of training, (2) control group (n=72): received no AMC training; assessment: Combined Raven, Qualification Examination of the Chinese Abacus and Mental Arithmetic Association.	The AMC-trained group had better math abilities. No significant differences were found in local or global efficiency or modularity. The local efficiency of the cingulo-opercular network was lower in AMC group. The visual network showed greater local efficiency and more intra-module connections in AMC group. Visuospatial neural mechanisms play an important role in AMC training.
Wang et al., 2019^16^	To investigate the effects of AMC training on arithmetic performance and VSWM in children.	n=144 participants (n=112 analyzed); children from first year of primary school; intervention length: 5 years; study groups: (1) AMC group (n=61): 2 hours AMC training per week; (2) control group (n=51): 2 hours per week of conventional calculation and reading; pre and post-intervention testing: Raven's Intelligence Test; post-intervention tests: 2 arithmetic tests (computerized mental calculation task and HRT) and a VSWM task; final sample for fMRI data statistics: 50 children.	Long-term AMC training improved arithmetic ability and had a potential positive effect on VSWM due to a transfer effect of AMC training.
Dong et al., 2016^32^	To examine whether AMC training is helpful to promote improvement in VSWM and verbal WM, and how VSWM-related cortical activity is altered by AMC training with a longitudinal design.	n=36 participants (n=33 analyzed); young university students with mean age of 21-22 years with no experience of calculating using a PA or MA; intervention length: 20 days; study groups: (1) abacus group (n=18): 90 min per day (1-day break), 2-3 days of physical abacus training, assessment of proficiency: national standard assessment of AMC (10^th^ level) every 4 days; (2) control group (n=15); pre and post-intervention assessments: assessment of verbal WM (DMS/LMS), assessment of VSWM and underlying neural correlates (visuospatial n-back task both outside and inside fMRI scanner).	Results suggest AMC training not only improves calculating skills but also has the potential to promote individuals' verbal WM and VSWM capabilities, which is associated with the functional plasticity of the common neural substrates.
Matías-Guiu et al., 2016^22^	To explore the applicability of a cognitive stimulation method based on abacus arithmetic in elderly people with and without cognitive impairment. Secondary endpoints were family satisfaction, caregiver burden, and the behaviour and cognition of patients.	n=20 participants, age over 65 years; intervention length: 5 weeks; study groups: (1) healthy individuals with MMSE score >24 (n=6), (2) patients diagnosed with MCI (n=6), (3) patients diagnosed with probable early stage AD (n=8). Pre- and post-intervention assessments: MMSE and Trail Making Test A and B, GDS, for AD patients, the Zarit caregiver burden scale was applied. Intervention: 10 sessions of 150 minutes 2x per week, mixed groups (1 participant from each diagnostic group) of 10 participants comprising: 60 minutes of abacus calculation, 10 minutes of mental calculation, 45 minutes of other cognitive activities and 35 minutes of relaxing and/or concentration activities.	Changes in MMSE scores were seen, although no changes were observed on the TMT-A and B tests, the Yesavage Geriatric Depression scale or the Zarit caregiver burden scale.

AMC: abacus-based mental calculation; HRT: Heidelberg Rechentest; MA: mental abacus; PA: physical abacus; MCI: mild cognitive impairment. AD: Alzheimer's Disease; EF: executive function; MMSE: Mini-Mental State Examination; GDS: Geriatric Depression Scale; fMRI: functional magnetic resonance imaging; DMS: Digit/letter Memory Span; SWM: spatial working memory; VSWM: visuospatial working memory; IADLs: Instrumental Activities of Daily Living; DMS/LMS: digit/letter memory span.

The present study found only a few articles addressing the use of the abacus as a cognitive training tool. The interventions used in all studies was the *abacus-based mental calculation* (AMC) technique, whereby participants first learn how to manipulate the abacus and, after achieving proficiency, perform calculations using a mental image of the device, manipulating the beads of the mental abacus (MA).^20^ One of the included studies^22^ also applied a task using a physical abacus (PA).

Seven studies showed an association between WM and AMC training.^16^ ^,^ ^17^ ^,^ ^28^ ^,^ ^29^ ^,^ ^30^ ^,^ ^31^ ^,^ ^32^ The one study in older adults^22^ showed that this type of cognitive intervention promoted significant improvement in MMSE scores.

## DISCUSSION

The objective of the present study was to carry out a systematic review of studies involving delivery of a cognitive training program using the abacus to stimulate target cognitive abilities. A total of 8 eligible studies were retrieved, whose objectives included investigating the effect of WM,^16^ ^,^ ^28^ ^,^ ^32^ arithmetic performance,^16^ ^,^ ^28^ ^,^ ^29^ short-term memory,^31^ executive functions and underlying brain activity^17^ and on brain networks,^28^ and to determine the applicability of abacus use as a method of cognitive stimulation for older adults.^22^

### Cognitive training using abacus in children

All 6 studies involving samples of children performed AMC interventions, in which a PA was used at only one stage to aid learning of AMC.^20^ ^,^ ^21^ ^,^ ^28^ ^,^ ^30^

Five articles showed that AMC training promoted improved arithmetic skills.^16^ ^,^ ^17^ ^,^ ^28^ ^,^ ^29^ ^,^ ^30^ This result is consistent with the classic studies conducted by Hatano et al.^33^ and Stigler,^34^ confirming that solving extremely complex arithmetic problems can be enhanced and accelerated by AMC training. According to Wang et al.,^20^ the MA is a tool that is commonly taught to Chinese children in order to enhance their academic performance.

Besides this effect, some studies have reported other positive results of AMC training in children. The results of the study by Wang et al.,^17^ involving 51 participants assessed over a 5-year period, suggested that this type of cognitive training promoted improvement in WM of the trained participants. This finding corroborates the result of the study by Bergman-Nutley and Klingberg,^35^ which applied WM training in 176 children and adolescents with deficit in this executive function. After 5 weeks of training, participants showed improvement in WM performance.

The study by Wang et al.^29^ of 70 children receiving AMC training for 3 years and the study by Wang et al.^16^ of 112 participants trained on AMC for 5 years reported an association between AMC training and visuospatial WM. Wang et al.^16^ explained this association may have occurred due to a transfer effect, i.e. AMC training confers, according to Barner et al.,^28^ potential changes in ability of the trained individual to create and manipulate structures in visuospatial WM. This result is in line with that suggested by Hu et al.^36^ in their study of 50 children, 25 of whom underwent previous AMC training. The authors used neuroimaging markers and analyzed possible changes in brain tissue resulting from AMC training. Their results, based on functional Magnetic Resonance Imaging (fMRI) study, showed that trained children can improve their memory ability and the integrity of brain areas recruited in motor and visuospatial processes.

In the study by Bhaskaran et al.^31^ of 100 participants over a 2-year period, AMC training in children led to improved short-term memory performance, more specifically visual and auditory memory. Confirming the findings of the classic study of Hatano and Osawa,^37^ and also of Tanaka et al.,^38^ the results of the study by Bhaskaran et al.^31^ showed that learners that became expert in the use of MA could memorize a very long sequence of numbers and letters, both for forwards and backward span, using the Letter-Number Sequencing WM test. The authors explained that, while memorizing in forward span is immediately associated with short-term memory performance, the mental processes involved in reversing the sequence can also reflect the use of visuospatial abilities.

By contrast, the study by Barner et al.^28^ of 183 children over a 3-year period, while revealing improvement in arithmetic tasks, reported that AMC training provided no benefit for basic cognitive abilities (including WM) and that individuals with better WM at the beginning would be better able to achieve greater proficiency in the use of a MA. The study of Xie et al.^30^ assessing 162 children for 1 year supported this finding, documenting that visuospatial neural mechanisms existing prior to intervention play an important role in MA learning by children. Barner et al.^28^ highlighted this is due to an effect called cognitive mediation, i.e. preexisting cognitive abilities served as modulators of abacus expertise.

Results of the majority of studies involving cognitive training using the abacus in children showed that this type of training promoted improvements predominantly in arithmetic skills, but also led to transfer effects for WM. However, it is noteworthy that the results of the study conducted by Dunning et al.^39^ of 94 children receiving WM training for 6 weeks led authors to conclude that benefits of this type of training may not extend to other cognitive abilities or to the development of academic abilities, beyond the structured WM tasks performed.

### Cognitive training using abacus in young adults

The study by Dong et al.^32^ used AMC training as an intervention tool in 33 young university students aged 21‒22 years with no experience of PA or MA use. The individuals underwent 20 days of training given in 90-minute sessions. The results showed that AMC training improved not only calculation abilities but also could potentially enhance WM abilities, both verbal and visuospatial. Visuospatial WM was more effective in trained individuals, since these subjects required less visuospatial attention when performing tasks involving this function. According to the authors, besides promoting WM abilities, AMC training can also serve as an intervention tool for individuals that display impairment in this function.

Similarly, the study by Barroso et al.^40^ also showed improvement in WM among young adults. The study assessed the effectiveness of cognitive training using electronic games for improving cognitive functions of attention and memory in university students aged 18‒29 years. A total of 25 students were selected and split as follows: one group was submitted to training for a period of 5‒8 weeks while a second group took part in the intervention for 9‒16 weeks. The results found by the authors revealed that the training had beneficial effects for WM of the group which underwent the highest number sessions.

The meta-analysis performed by Karbach and Verhaeghen^14^ assessed 49 studies involving young and older adults, examining the effects of cognitive training on the domains of executive function and WM. The authors showed that interventions focused on WM had a positive impact for the participants that underwent the training, revealing better performance relative to the control group for both target and proximal transfer tasks.

Conversely, the findings of the study conducted by Thompson et al.,^41^ in which young adults aged 18‒45 years completed 20 sessions of a WM training program, failed to observe transfer for untrained tasks upon comparing the trained group with the control group. This result implies that the benefits of training were limited to the target task.

Overall, the findings of the studies in young adults analyzed in this review, with regard to the relationship between AMC and WM, are consistent with the results found for the studies in children. This suggests that AMC training is applicable for improving cognitive abilities in populations of different age strata.

### Cognitive training using abacus in older adults

The study by Matías-Guiu et al.,^22^ cited previously, used both AMC and PA training interventions. Of the studies included in the present review, this was the only investigation centered on the older population. The study assessed a total of 20 individuals aged ≥65 years with the following characteristics: healthy elderly (n=6) (no cognitive impairment), elderly subjects diagnosed with MCI (n=6), and elderly diagnosed with early-stage AD (n=8), allocated into mixed groups during the course of the program.

The study results revealed improvement in general performance on the MMSE, suggesting a change in cognition after participating in the intervention. An association between better performance on the MMSE and memory training in healthy elderly was also found in the study of Irigaray et al.,^42^ confirming the positive effect of training for global cognitive functions. In the study by Matías-Guiu,^22^ no significant changes were evident in scores on tests measuring executive function or depression symptoms. The authors suggested that cognitive training using the PA and MA is valid for the older persons with or without cognitive impairment and can lead to improvement in global cognitive functions.

Unlike the other studies analyzed in this review, with respect to the association between the use of the abacus and WM, the study by Matías-Guiu et al.^22^ appeared to have methodological weaknesses in that instruments for assessing the target abilities were not employed. It can be inferred that the improvement in MMSE performance was due to gains derived from the transfer effect, a notion in agreement with the findings of the study by Brum et al.^43^

 In the study,^43^ the authors compared the efficacy of a WM training protocol offered in an individual (original) versus group (adapted) format. Older adults aged over 60 were assessed in two groups, performing tests in 2 waves of 3 sessions. For the first wave, participants were randomized into individual training (n=11) and individual control conditions (n=15). In the second wave, participants were randomized into group training (n=16) and group control conditions (n=17). According to the authors, findings showed that participation in training sessions (individual or group) had a positive impact on the target task and a transfer effect. However, participants who performed the activities individually had higher scores than those performing tasks in a group. These gains were evident at post-test and at follow-up after 6 months.

Borella et al.,^44^ in a study carried out in Italy, showed the efficacy of WM training in older adults for a target task (visuospatial WM) and on transfer tasks, with improvements in processing speed, inhibition, verbal WM, short-term memory and reasoning. There was evidence of maintenance of gains in the experimental group after 8 months.

In the study by Netto et al.,^45^ carried out in Brazil, the authors recruited a group of 20 healthy elderly and implemented a WM training program. Participants were divided into 2 groups: a training group (n=11) and an active control group (n=9) (socialization group). Participants were submitted to 12 training sessions consisting of explanations on theory (in which the concepts of WM and cognitive reserve were presented) and a practical part (exercises related to visual and auditory WM, such as mental calculations and games involving changing position of objects). The socialization groups took part in meetings discussing a range of topics, such as gastronomy and sports. At post-test, the authors found increased scores in the training group for performance on tests of focused attention and both episodic and short-term memory. The performance of the control group improved on tests of attention and episodic memory. The authors concluded that the WM training intervention appeared to have greater impact on cognitive tests compared to the socialization intervention. On the other hand, this impact occurred in a partial manner, in which no improvement was seen on tasks measuring the central executive.

The findings of the meta-analysis by Sala and Gobet also warrant mention.^46^ The authors analyzed the empirical evidence for the effects of 5 types of cognitive training programs on general cognitive ability (GCA), including WM training. The analysis of the studies suggested that the effects of WM training on GCA in older adults are minimal and limited to the trained task. At best, these interventions boosted performance on tasks similar to the trained task, showing that cognitive training does not enhance GCA.

Considering the impact of WM training for older adults and the association between AMC training and WM observed in studies involving other age groups, it follows that AMC training may also promote WM gains in the older population. In addition, given that the study in younger adults^32^ revealed the effectiveness of AMC training as an intervention tool for individuals with WM deficits, it can be assumed that older adults with the condition can also benefit from AMC training as a form of WM rehabilitation.

### Considerations on cognitive training and the benefits of non-pharmacological interventions

Cognitive plasticity turns out to be an important field for clinical research, as it brings forth essential information about human aging,^26^ ^,^ ^47^ ^,^ ^48^ and also fundamental implications for the clinical practice of the gerontologist, as the optimization of memory is related to the health, autonomy and independence of older persons.

However, it is observed that cognitive training studies differ not only in terms of duration but also, in particular, in terms of the strategies taught and the methodology used, with great diversity in the literature regarding the magnitude of the cognitive benefits, the generalization for untrained tasks and the maintainability of long-term training.^49^ ^,^ ^50^ ^,^ ^51^

Through this review, it was observed that new designs of cognitive interventions are proving to be innovative, such as studies with training on the use of abacus, which has been adopted in elementary and high schools as tools to optimize the learning process of children and adolescents. Improvements in cognitive variables and in neuroimaging markers were found, reinforcing the hypothesis of cognitive plasticity.^16^ ^,^ ^31^ ^,^ ^32^ However, in healthy older adults and elderly people, as described in this literature review, a very small number of studies were found.

The integrative literature review carried out by Costa et al.^52^ indicates that motor intervention, cognitive intervention and music therapy have shown to be promising non-pharmacological interventions for the treatment of patients with AD. The authors found that the literature presents evidence of the impacts of cognitive intervention, providing patients undergoing this practice with significant effects on cognition and on the performance of ADLs, as well as on the stabilization of clinical symptoms. The authors also point out that combined interventions that stimulate motor and cognitive functions can be a viable and beneficial option in non-pharmacological treatment, reducing dependence when performing ADL's and improving the quality of life of individuals.

As previously described, the AMC technique makes impacts related to the improvement of cognitive skills, such as WM, visuospatial WM and on the general cognitive performance of people of different age groups. In addition, the training phases with PA can promote the training of motor skills. Thus, the impacts of training in AMC and with PA are in line with those reported by Costa et al.^52^ and are also characterized as an effective non-pharmacological intervention.

The studies included in this review reported cognitive performance gains in individuals of different age groups after participating in cognitive training programs using an abacus. Therefore, considering the promotion of health and quality of life of the older population in terms of cognitive ability and all its related aspects, highlighting the applicability of AMC training programs during all phases of one's development is highly relevant.

A limited number of articles addressing the theme was identified, most of which carried out with children, indicating that the use of abacus as a cognitive training tool is prevalent in this age group, which represents a limitation of the present study. In addition, as it is a traditional tool of some Eastern countries, inserted in the culture and daily life of this population, the studies analyzed in this review are mostly performed in countries with these origins. Thus, it is possible to point out as another limitation of this study the scarcity of researches that carry out this type of intervention in other populations, evidencing the need for future studies in this perspective.

A limitation of the present study was the dearth of studies with well-designed methods, including the application of sensitive specific tests for measuring performance of cognitive training using an abacus, showing that you need research with more criteria methods.

Another relevant finding of the present study concerning the results of the initial search was that, out of the 22 studies retrieved, only one focused on the elderly population, which indicates the need of carrying out a greater amount of research on cognitive training with older persons and with the abacus as a training tool, in different cultures, aiming to confirm whether the findings described for younger individuals are generalized to the cognitive skills of the older persons. Cognitive training is a potential intervention of cognitive reserve optimization.

When considering the growth of the elderly population and neurodegenerative diseases, which accompany the aging process, investing in non-pharmacological cognitive interventions, such as the use of abacus, can be a good strategy for promoting health, quality of life and well-being. The present study also highlights the research gap in this area, indicating a problem for future studies to address. It is also suggested for future studies that they present in their methodological design interventions with the use of the abacus with an emphasis on healthy older adults and elderly people.
